# Photocatalytic degradation of organic dyes: Pd-γ-Al_2_O_3_ and PdO-γ-Al_2_O_3_ as potential photocatalysts

**DOI:** 10.1039/d0ra10290c

**Published:** 2021-02-04

**Authors:** Avvaru Praveen Kumar, Dinesh Bilehal, Aschalew Tadesse, Deepak Kumar

**Affiliations:** Department of Applied Chemistry, School of Applied Natural Science, Adama Science and Technology University PO Box 1888 Adama Ethiopia drkumar.kr@gmail.com; Department of Chemistry, Karnatak University Dharwad-560008 Karnataka India; Department of Pharmaceutical Chemistry, School of Pharmaceutical Sciences, Shoolini University Bajhol Solan H.P-173229 India

## Abstract

This work describes photocatalytic application of γ-alumina (γ-Al_2_O_3_) surface-anchored palladium and palladium oxide nanoparticles (Pd-γ-Al_2_O_3_ and PdO-γ-Al_2_O_3_ NPs) synthesized by a novel co-precipitation technique. The palladium(0) NPs (Pd-γ-Al_2_O_3_) were formed by calcination of the sample in inert medium, whereas PdO NPs (PdO-γ-Al_2_O_3_) were obtained by calcination of the sample in atmospheric oxygen. As-synthesized Pd-γ-Al_2_O_3_ and PdO-γ-Al_2_O_3_ NPs are characterized by X-ray diffraction, Fourier transform-infrared spectroscopy, field emission scanning electron microscopy and photoluminescence (PL) spectra. The PL spectra of Pd-γ-Al_2_O_3_ and PdO-γ-Al_2_O_3_ NPs display visible-light emissions from 450 to 500 nm at room temperature. This work aims to study the photocatalytic degradation of organic dye pollutants, including bromocresol green (BCG), bromothymol blue, methylene blue and methyl orange using Pd-γ-Al_2_O_3_ and PdO-γ-Al_2_O_3_ NPs as potential photocatalysts. Experimental parameters, including the admitting concentration of the organic dye solution, Pd-γ-Al_2_O_3_ and PdO-γ-Al_2_O_3_ photocatalyst dosage, and pH, were varied to ascertain favorable conditions for photocatalytic degradation. The results indicate that the organic dye BCG is completely (100%) degraded in aqueous solution under ultraviolet light, compared with the other organic dyes. Furthermore, Pd-γ-Al_2_O_3_ NPs showed better photocatalytic performance than PdO-γ-Al_2_O_3_ NPs. The possible photocatalytic degradation mechanism of the organic dyes by Pd-γ-Al_2_O_3_ and PdO-γ-Al_2_O_3_ photocatalysts is proposed. The studies reveal that Pd and PdO NPs anchored on the γ-Al_2_O_3_ surface are promising and effective catalysts for photocatalytic degradation of organic dyes.

## Abstract 2

Mechanism of photocatalytic degradation of organic dyes by Pd/PdO NPs.

## Graphical abstract

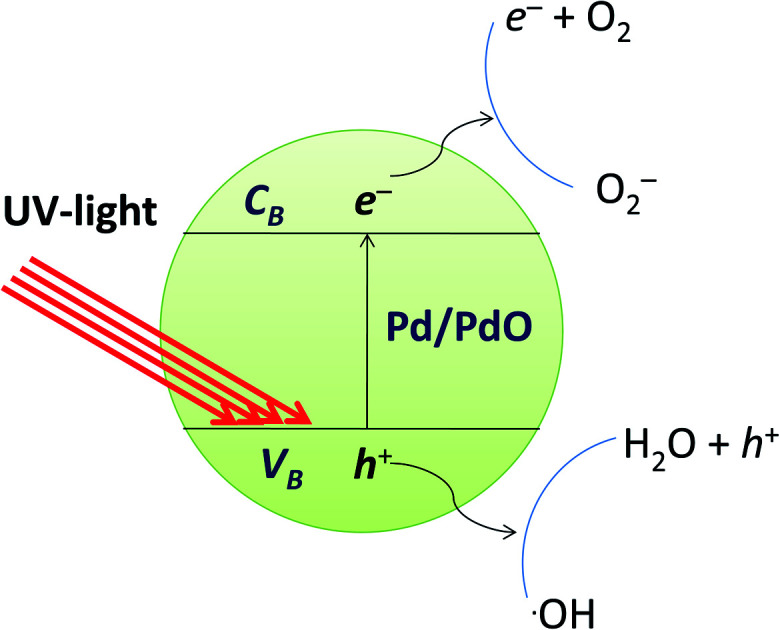

## Introduction

There has been much attention on metal nanoparticles (NPs), which have contributed to the field of nanoparticle research. Transition metal NPs exhibit significant features in their structural arrangement and the elucidation of various applications. In particular, nano-sized transition metals have received much attention from basic science as well as from technological perspectives, due to the quantum size effect resulting from the reduction of free electrons.^1^ Research into the preparation, structural determination and exploration of several applications of these nano systems has been undertaken. Thus, transition metal nanomaterials are prepared in many different forms, such as spherical shapes, nanorods, nanowires, nanotubes, nanowhiskers, nanohorns, nanopyramids and other nano-composites, based on features of interest or the requirements of the application being studied.^2–4^ Moreover, due to their characteristic properties they are strikingly different from the bulk material.

Transition metal nanoparticles have been studied in several fields, such as catalysis, optics, biosensors, chiral separation, magnetic devices, solar cells, electronics and adsorbents.^5–8^ The molecules in NPs are small and the percentage of molecules on the surface is high, and this property means that the NPs are very interesting for applications in catalysis.^9–11^ The use of noble transition metal NPs as catalysts is limited by their scarcity and high cost. Therefore, it is important to improve their catalytic performance and reduce their cost. This has been studied in several ways by altering the materials to enhance their activity, stability, selectivity of active sites and surface area, and thus reduce costs.^12^ The size and shape of metal NPs has proven them to be attractive for their increased and improved catalytic activity and further modification of these materials is of interest.

NP-based noble metals have been developed and successfully applied in different fields, such as catalytic activity, storage of hydrogen, wastewater treatment and organic synthesis, *etc.* This is mainly because of they possess excellent thermal and mechanical strength, coordination sites, highly ordered structure and high specific surface area.^13–15^ The noble metals, gold (Au), platinum (Pt), palladium (Pd) and silver (Ag) are used to differentiate electron–hole pair recombination by acting as electron acceptors and also promote the interfacial charge transfer process.^16^ Among the noble metals, Pd and Pd-based NPs play a significant role in many applications.^17^

Photocatalytic dye degradation is advantageous due to the pollutant being degraded rather than transformed under atmosphere conditions. This method allows a large variety of environmental toxins, including pesticides, herbicides and endocrine disruptors, to be eliminated. Although amazing work has been performed with different photocatalysts on a laboratory scale using metals and metal oxides, it does require some improvements for use on an industrial scale. However, many factors, such as large band gap and lack of reliable and cost-effective photocatalysts, restrict the implementation of this procedure. In order to better understand the photocatalytic process and its applications, further study is needed to explore the degradation of real-water contaminants.

The Pd NPs have largely been applied as catalysts because Pd NPs possess a low Fermi energy level and high work function (∼5.12 eV). The photocatalytic reaction is an advanced oxidation process that has been widely examined in several fields. The evolution of H_2_ through water splitting and degradation of organic pollutants^18,19^ are the main, significant applications. Photocatalysis is also an effective alternative to remove dyes and organic pollutants from water and industrial waste. In recent years some research has been published on the photocatalytic activity of Pd-based nanomaterials synthesized by different methods. Özacar *et al.* compared Pd/zinc oxide (Pd/ZnO) photocatalysts synthesized through various Pd-doping methods for degradation of Congo red.^20^ In their work, Pd/ZnO synthesized by a borohydride reduction method showed good photocatalytic activity. J.-H. Lee *et al.*^21^ reported the effect of Pd NPs on the photocatalytic characteristics of N-doped titania catalysts, and these were prepared *via* anodizing, hydrazine hydrate treatment and photoreduction of Pd ions. Roy's research group^22^ developed an environmentally friendly approach to synthesize polyaniline-supported Pd catalysts for the reductive degradation of organic dyes, including methyl orange, rhodamine B and methylene blue, in the presence of the reducing agent sodium borohydride. In addition, Pd NPs were fabricated *via* ultrasonication of Andean blackberry leaf extract and were used for photocatalytic degradation of methylene blue.^23^ Rao *et al.*^24^ reported enhanced photocatalytic degradation of bisphenol A by Pd/PdO/β-Bi_2_O_3_ microspheres and M. A. Khan's research team^25^ used guar gum as stabilizer to synthesize Pd NPs and applied the Pd NPs as catalysts for the degradation of azo dyes. However, these methods are complicated and difficult for synthesizing Pd NPs and some require sophisticated equipment. Amongst the synthesis methods for NPs, co-precipitation is a successful method for obtaining a high degree of homogeneity in combination with small-particle size and narrow particle size distribution, to achieve high catalytic activity.

Kumar *et al.*^26^ developed a co-precipitation method to synthesize Pd and PdO NPs on alumina surface and their catalytic application of Hiyama cross-coupling, Suzuki coupling, aerobic oxidation reactions and alkene and alkyne hydrogenation. In the present work, we discovered an economic and convenient method for elimination of organic dyes, including bromocresol green, bromothymol blue, methylene blue and methyl orange from water using Pd and PdO NPs anchored on the alumina (γ-Al_2_O_3_) surface (Pd-γ-Al_2_O_3_ and PdO-γ-Al_2_O_3_) as promising photocatalysts. Until now, there has been no report of research on the use of Pd-γ-Al_2_O_3_ and PdO-γ-Al_2_O_3_ NPs for photodegradation of dyes under ultraviolet (UV) light.

## Experimental

### Materials

Aluminum(iii) nitrate nonahydrate (Al(NO_3_)_3_·9H_2_O) and palladium(ii) chloride (PdCl_2_) were obtained from Sigma-Aldrich (St. Louis, MO, USA). Ammonium hydroxide (NH_4_OH) was purchased from Sigma. Organic dyes, bromocresol green (BCG), bromothymol blue (BTB), methyl orange (MO) and methylene blue (MB), were received from Sigma-Aldrich. HPLC-grade distilled water was used throughout. Other chemical reagents used were of analytical reagent grade with greater than 99% purity, and were used as obtained without further purification.

### Synthesis of Pd/PdO NPs on the alumina surface

The Pd and PdO NPs on the γ-alumina surface were synthesized by chemical co-precipitation using our previously reported method.^26^ Briefly, the Pd and PdO NPs were synthesized from three different concentrations of PdCl_2_, 0.01 M, 0.025 M and 0.05 M, while keeping the Al(NO_3_)_3_·9H_2_0 (0.1 M) concentration constant. All concentrations were prepared separately in individual standard flasks. The individual precursor solutions were mixed and stirred, with the addition of excess NH_4_OH solution to obtain a pH of 9.0. Then, stirring was continued for 2 hours to obtain a uniform dispersion of particles. The suspension obtained was centrifuged to give a gelatinous precipitate. The precipitate was rinsed with H_2_O and dried at 120 °C in an oven. The dried precipitate was ground in a mortar and calcined in a furnace at 600 °C for 2 hours under atmospheric oxygen to obtain PdO-γ-Al_2_O_3_ NPs, and in an inert medium (nitrogen atmosphere) to obtain Pd-γ-Al_2_O_3_ NPs.

### Characterization techniques

A Philips X'Pert MPD 3040 diffractometer was used to obtain X-ray diffraction (XRD) patterns for the Pd-γ-Al_2_O_3_ and PdO-γ-Al_2_O_3_ NP calcinated samples. A Nicolet FT-IR 400 spectrophotometer (Nicolet iS10, SCINCO, USA) was used to record the Fourier transform-infrared (FT-IR) spectra using the KBr pellet method for preparation of the FT-IR samples. Field emission scanning electron microscopy (FE-SEM; MIRA II, LMH) operated at an acceleration voltage of 20 kV was performed to capture surface images of the calcined samples of Pd and PdO NPs anchored on the γ-Al_2_O_3_ surface. The elemental compositions of the calcined NP samples were determined using energy-dispersive X-ray analysis (EDAX) spectroscopy. The fluorescence emission and excitation spectra were assessed using a Shimadzu, RF-5301PC spectrofluorophotometer with a xenon lamp (150 W) as the excitation source. A Lambda 950 spectrometer (PerkinElmer) was used to record the UV-visible (UV-vis) spectra of the samples at wavelengths of 200–800 nm.

### Photocatalytic degradation

The photocatalytic activity of Pd-γ-Al_2_O_3_ and PdO-γ-Al_2_O_3_ NPs was studied by degradation of individual aqueous solutions of MB, MO, BTB and BCG dyes under UV light. A 10 mL portion of dye solution of known concentration and a specific amount (0.1 g) of Pd-γ-Al_2_O_3_ or PdO-γ-Al_2_O_3_ were mixed in a beaker. The beaker contents were stirred magnetically for 60 min in the absence of light in order to reach adsorption–desorption equilibrium between the Pd-γ-Al_2_O_3_ or PdO-γ-Al_2_O_3_ photocatalysts and the organic dyes. Finally, the sample solutions were placed under UV light (UV cabinet). A mercury lamp (PHILIPS, TUV 8 W T5, *E*_max_ = 254) with UV light intensity of 4 mW cm^−2^ was used for illumination of the photocatalyst to produce highly reactive species. The UV light intensity was measured with the help of an optical power meter (Newport 2936-C) to examine the photocatalytic degradation of the organic dyes. The progress of the photodegradation reaction was monitored at different time intervals by measuring the absorbance, at specific wavelength, of the organic dyes using UV-vis spectrophotometry.

## Results and discussion

The Pd-γ-Al_2_O_3_ and PdO-γ-Al_2_O_3_ NPs on the γ-alumina surface were synthesized by a chemical co-precipitation method in which the individual precursor solutions of Pd salt of three different concentrations and aluminium nitrate solution of constant concentration were mixed. The resultant solution was maintained at pH of 9.0 by the addition of NH_4_OH solution while stirring to obtain Pd-dispersed alumina material. The obtained product was calcined in atmospheric oxygen to obtain PdO-γ-Al_2_O_3_ NPs, and calcined in inert medium (nitrogen atmosphere) to obtain Pd-γ-Al_2_O_3_ NPs. The formation Pd and PdO NPs on the γ-Al_2_O_3_ surface was confirmed by XRD analysis and the results obtained are similar to our previous report.^26^

### FT-IR spectroscopy

**Fig. 1:**
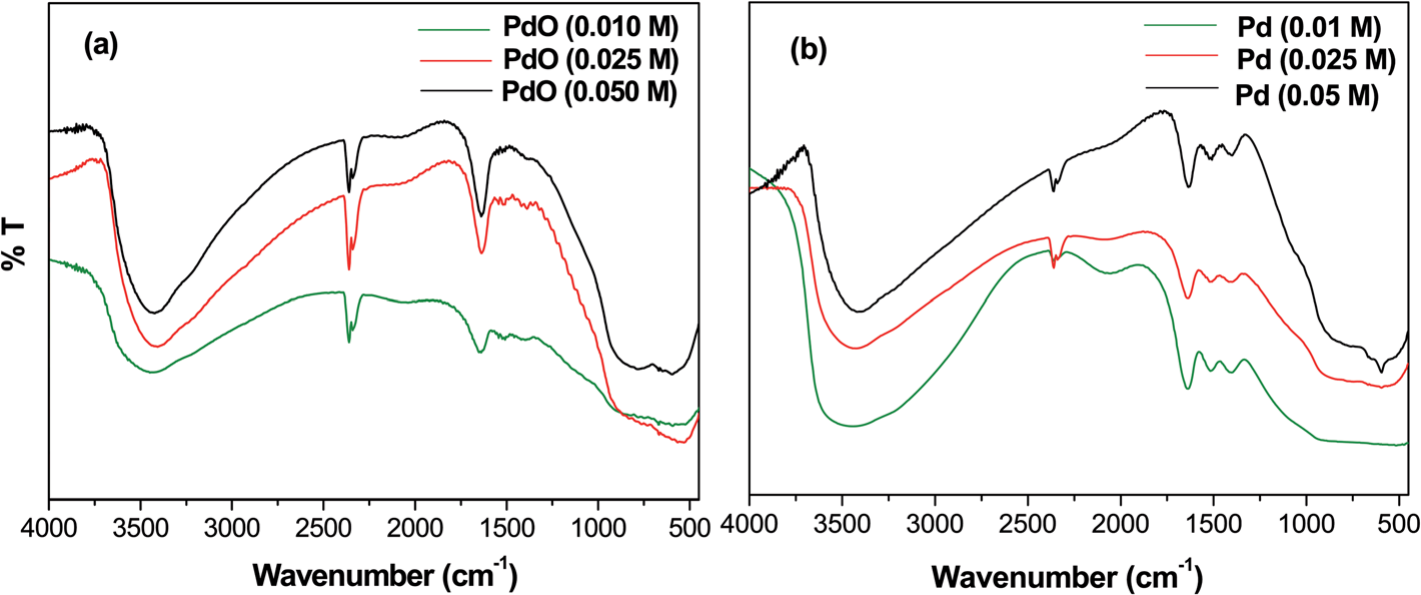
FT-IR spectra of (a) PdO-γ-Al_2_O_3_ NPs and (b) Pd-γ-Al_2_O_3_ NPs.

The FT-IR spectra of Pd-γ-Al_2_O_3_ and PdO-γ-Al_2_O_3_ NPs were measured from 4000 to 400 cm^−1^ and are shown in Fig. 1. A wide absorption band from 3400–3450 cm^−1^ is observed, which is attributed to O–H stretching in Pd–OH groups, or physiosorbed H_2_O.^27^ A bending peak detected at around 2300 cm^−1^ is assigned to the CO_2_ absorption,^28^ and an absorption frequency at 1610 cm^−1^ has been confirmed to be H–O–H.^29^ The presence of these two bands (CO_2_ and absorbed H_2_O) is due to absorption of moisture during testing of the sample. Two new bands, found at 550 and 600 cm^−1^, are expected to be distinctive for the M(Pd/Al)–O stretching frequencies of the Pd–O and Al–O vibrations for the Pd/PdO NPs on the γ-Al_2_O_3_ surface.^30^

### FE-SEM

**Fig. 2:**
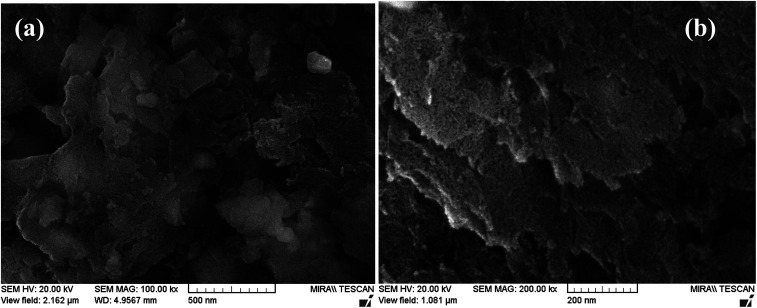
FE-SEM images of (a) Pd-γ-Al_2_O_3_ and (b) PdO-γ-Al_2_O_3_ NPs.

**Fig. 3:**
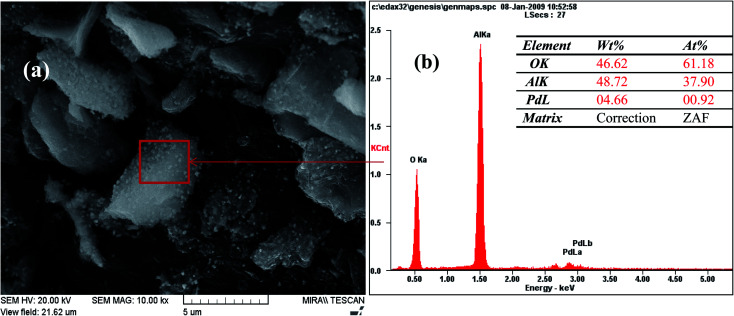
EDAX analysis of Pd-γ-Al_2_O_3_ NPs.

The FE-SEM images of Pd-γ-Al_2_O_3_ and PdO-γ-Al_2_O_3_ NPs anchored on the γ-Al_2_O_3_ surface are presented in Fig. 2. From the SEM images it can be seen that the Pd NPs, as well as the PdO NPs, are fused in the γ-Al_2_O_3_ matrix during the process of calcination.^26^ The sizes of the Pd and PdO NPs are between 10 and 30 nm and the sizes of the NPs vary with the Pd salt concentration. The average distribution of the NPs increases with increase in concentration of the Pd salt. The shape of the Pd and PdO NPs has been observed to be spherical.^26^ It should be noted that in order to minimize the surface energy of the particles, the particle shape would be spherical.^31,32^ So, the size distribution becomes narrower with growth of the spherical templates. Furthermore, EDAX analysis was performed in conjunction with FE-SEM to support the existence of Pd and PdO NPs in the γ-Al_2_O_3_ matrix. The EDAX analysis of Pd-γ-Al_2_O_3_ NPs is presented in Fig. 3.

### Photoluminescence spectroscopy

**Fig. 4:**
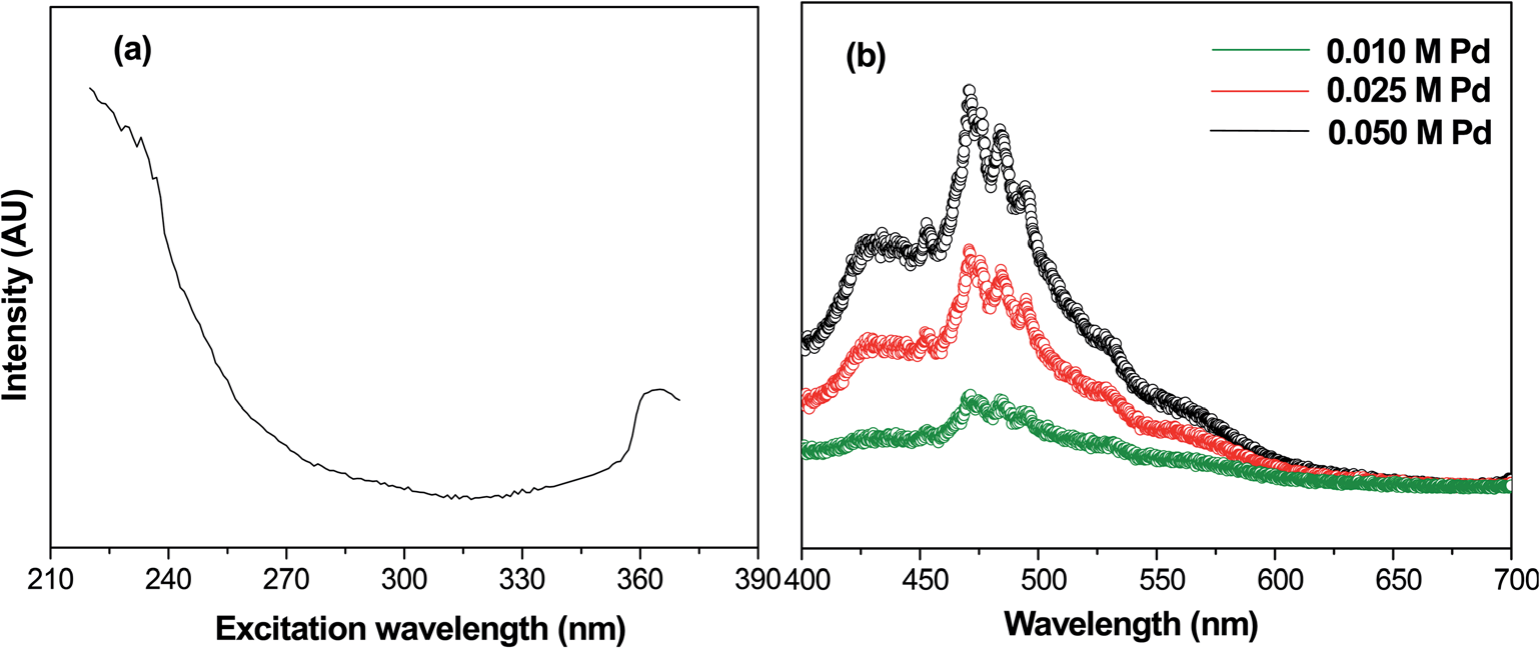
(a) Fluorescence excitation spectrum of PdO-γ-Al_2_O_3_ NPs at wavelength 235 nm. (b) Photoluminescence spectra of PdO-γ-Al_2_O_3_ NPs.

Photoluminescence spectroscopy is generally used to characterize the surface processes for photogenerated electron–hole pairs.^33^Fig. 4(a) shows the fluorescence excitation spectra of calcined samples of PdO NPs on the γ-Al_2_O_3_ surface at a wavelength of 235 nm. In this fluorescence excitation spectrum, the highest intensity peak was observed at 365 nm, which represents the maximum transition probability/resonant absorption. This maximum intensity excitation peak at 365 nm is due to the transition between the first excited state (E_1_) and the ground state (E_0_). This transition belongs to the 4d electrons of palladium, since the noble metals exhibit fluorescence through transition of electrons in the band below the Fermi level to holes in the d bands.^34,35^ The photoluminescence spectra of Pd-γ-Al_2_O_3_ NPs recorded at 365 nm are shown in Fig. 4(b). It is known that the photoluminescence emission is due the recombination of charge carriers. From Fig. 4(b), it can be clearly observed that Pd-γ-Al_2_O_3_ NPs showed fluorescence emissions at 453, 472, 484 and 494 nm. The variation in the photoluminescence emission intensity was associated with the recombination rate of the excited electron and hole pairs. As the concentration of the palladium varies, the intensity of the peaks also differs. A weak/low photoluminescence emission intensity was identified for low Pd concentration samples, indicating a weak/low recombination rate due to the fact that more electrons are being trapped/transferred. Moreover, it was revealed that Pd NPs deposited on the surface of the γ-Al_2_O_3_ increased the transfer/trapping of electrons, thereby overcoming the recombination rate. This high efficiency for charge carrier separation increases the lifetime of the reactive electron–hole, and thereby leads to improved photocatalytic performance of the nanophotocatalysts Pd-γ-Al_2_O_3_ and PdO-γ-Al_2_O_3_.

### Photocatalytic degradation of organic dyes

**Fig. 5:**
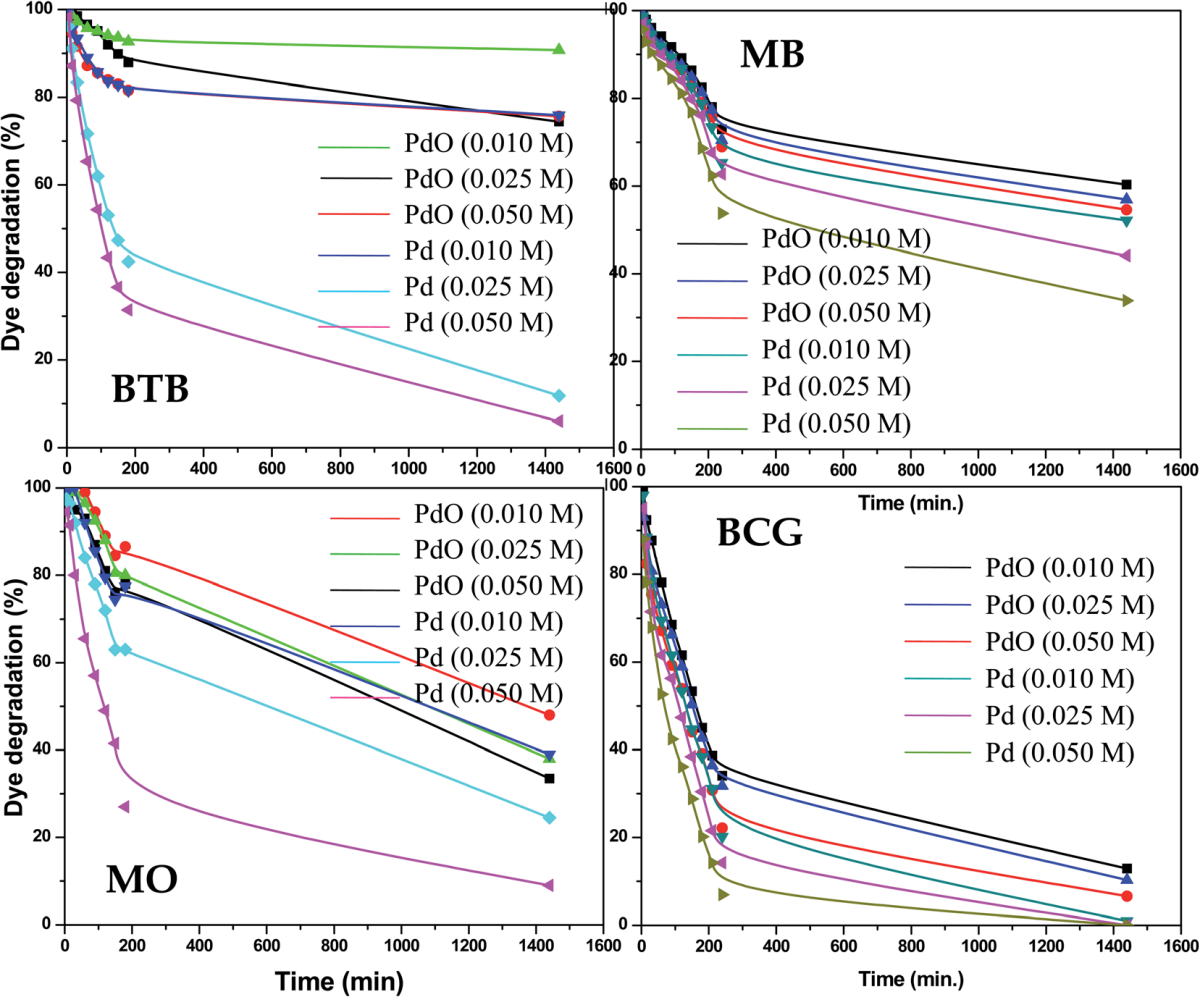
Percentage photodegradation of organic dyes by Pd-γ-Al_2_O_3_ and PdO–Al_2_O_3_ catalysts.

The photocatalytic activity of the as-synthesized Pd-γ-Al_2_O_3_ and PdO-γ-Al_2_O_3_ NPs was investigated for photodegradation of four organic pollutant dyes (BCG, BTB, MB and MO) under UV-light irradiation. The photocatalytic activity of Pd-γ-Al_2_O_3_ and PdO-γ-Al_2_O_3_ NPs was monitored by measuring the maximum absorbance intensity (*λ*_max_) of the organic dyes BCG, BTB, MB and MO at 423, 427, 664 and 464 nm, respectively. Fig. 5 shows the photocatalytic performance of the Pd-γ-Al_2_O_3_ and PdO-γ-Al_2_O_3_ NPs for degradation of BCG, BTB, MB and MO under UV-light irradiation. The following equations were employed for estimation of the percentage degradation of the organic dyes in aqueous medium:Degradation rate (%) = (*C*_0_ − *C*_*t*_/*C*_0_) × 100Degradation rate (%) = (*A*_0_ − *A*_*t*_/*A*_0_) × 100where, *C*_*t*_ is concentration of the dye solution after UV irradiation at time *t*, *C*_0_ is the initial concentration of the dye solution, *A*_0_ is the initial absorbance of the dye solution and *A*_*t*_ is the absorbance of the dye solution after UV-light irradiation at time *t*.^36^

The Pd-γ-Al_2_O_3_ and PdO-γ-Al_2_O_3_ NPs as photocatalysts were examined for photodegradation of four organic pollutant dyes, BCG, BTB, MB and MO under constant experimental conditions (degradation time from 0 to 1440 min (24 hours)). A control experiment was also carried out to check that there was no degradation of organic dye in the absence of Pd-γ-Al_2_O_3_ and PdO-γ-Al_2_O_3_ photocatalysts and it was observed that there was no, or very weak, photodegradation in the absence of the photocatalysts. The time course degradation curves of BCG, BTB, MB and MO under UV light (Fig. 5) indicate that organic dye degradation increases by increasing the time of UV-light irradiation. Furthermore, the photocatalytic degradation results reveal that the degradation for all organic pollutants mostly takes place within 240 min (4 hours). Complete degradation (100%) of organic pollutant BCG was observed using all different concentrations of Pd-γ-Al_2_O_3_ NPs by increasing the UV-light irradiation time, while PdO-γ-Al_2_O_3_ NPs were degraded by 88–93%. For the other organic dyes, BTB, MB and MO, a photodegradation of 94%, 65% and 91%, respectively, was achieved using Pd-γ-Al_2_O_3_ NPs, while a degradation of 26%, 46% and 68%, respectively, was found with PdO-γ-Al_2_O_3_ NPs. From these results it has been confirmed that Pd-γ-Al_2_O_3_ and PdO-γ-Al_2_O_3_ NPs showed higher photocatalytic efficiency under UV-light irradiation. This indicates that Pd-γ-Al_2_O_3_ and PdO-γ-Al_2_O_3_ NPs have a higher electron–hole pair formation tendency and therefore higher formation of superoxide and hydroxyl radicals. These findings demonstrate that the photocatalytic reaction was initiated and caused by the irradiation effect on the photocatalysts. A comparative study of photocatalytic degradation of organic dyes (MO, MB, BCG and BTB) by various photocatalysts from a literature survey is shown in Table 1. Our current study shows that the synthesized Pd-γ-Al_2_O_3_ and PdO-γ-Al_2_O_3_ NPs are promising candidates for removal of organic dyes.

**Table 1:** Comparison of the photodegradation of organic dyes using different photocatalysts

Photocatalysts	Preparation method	Light irradiated	Pollutant	Lamp power (W)	Catalyst dose	Degradation (%)	Ref.
Fe_2_O_3_/TiO_2_	UV-assisted thermal	UV	MO	9	As a slurry	61.5	37
TiO_2_-GO	Solvothermal	UV	MO	40	20 mg/50 mL	84	38
Cu-doped TiO_2_/ZnO	Sol–gel	Visible	MO	18–23	1.5 g L^−1^	85.5	39
PTA/ZR13	—	UV	MO	50	25 mg/100 mL	82	40
Cu-doped TiO_2_/ZnO	Sol–gel	Visible	MB	18–23	1.5 g L^−1^	73.2	39
CA–CNT/TiO_2_–NH_2_ composite nanofibers	Electrospinning technique	UV	MB	40	—	70	41
PTh-rGO-TiO_2_ nanocomposite	—	Visible	MB	—	0.25 mg mL^−1^	63	42
PTA/ZR31	—	UV	MB	50	25 mg/100 mL	87	40
PTA/ZR13	—	UV	BCG	50	25 mg/100 mL	89	40
ZnO nanodisks	Precipitation	UV	BCG	—	0.10 g	44.4	43
Co-ATC	—	UV	BCG	15	0.02 g/50 mL	40.5	44
Se NPs	—	UV	BTB	15	64 μg mL^−1^	62.3	45
PPT/PANI	—	UV	BTB	—	20 mg/50 mL	74	46
POM–ZrO_2_	Sol–gel	UV	BTB	400	20 mg/10 mL	52	47
Pd-γ-Al_2_O_3_ and PdO-γ-Al_2_O_3_	Chemical co-precipitation	UV	MO	8	0.1 g mL	94	Present method
			MB			65	
			BCG			100	
			BTB			91	

### Effect of Pd-γ-Al_2_O_3_ and PdO-γ-Al_2_O_3_ photocatalyst concentration

**Fig. 6:**
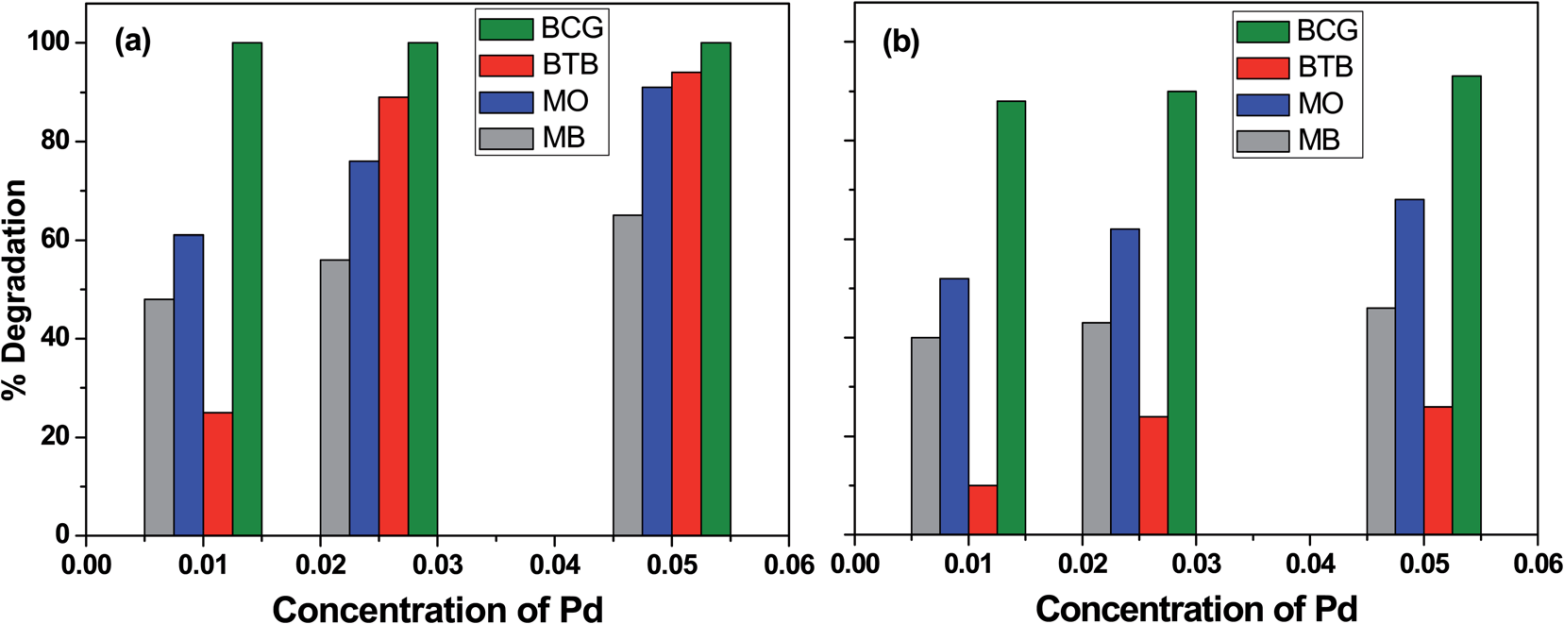
Percentage photocatalytic degradation of organic dyes at different concentrations of (a) Pd-γ-Al_2_O_3_ and (b) PdO-γ-Al_2_O_3_ catalysts.

The influence of photocatalyst concentration on the degradation of organic pollutants was tested by applying three different concentrations (0.010, 0.025 and 0.050 M) of the Pd-γ-Al_2_O_3_ and PdO-γ-Al_2_O_3_ photocatalysts under similar experimental conditions. Fig. 6 shows the comparison of the percentage photocatalytic degradation of the organic dyes at different concentrations of Pd-γ-Al_2_O_3_ and PdO-γ-Al_2_O_3_. As shown in Fig. 6, the photocatalytic degradation of the organic dyes increased with increasing concentration of both Pd-γ-Al_2_O_3_ and PdO-γ-Al_2_O_3_. This suggests that an increased amount of photocatalyst exposes a very active surface area and so increases the number of active sites on the photocatalyst surface, which increases the production of hydroxyl and superoxide radicals and provides more effective interaction of the substrate.^48^ It has been observed that the photocatalytic activity decreases at a concentration of Pd that is greater than 0.050 M. This is due to the increase in density of the solution with increased concentration of Pd, and possible aggregation of the Pd-γ-Al_2_O_3_ and PdO-γ-Al_2_O_3_ photocatalysts. This results in reduction of light transmission through the solution, causing a decrease in the photocatalytic degradation rate.^49^

Complete (100%) photocatalytic degradation of BCG organic dye was achieved using Pd-γ-Al_2_O_3_ photocatalysts at all three concentrations. The 0.010 M Pd concentration for the PdO-γ-Al_2_O_3_ photocatalyst degraded 88% of the BCG dye, and photodegradation was enhanced to 93% with an increase in the amount of Pd to 0.05 M. The other organic dyes, BTB, MB and MO, degraded by 25%, 48% and 61%, respectively, using 0.010 M Pd concentration for the Pd-γ-Al_2_O_3_ photocatalyst, and reached a maximum photocatalytic degradation of 94%, 65% and 91%, respectively, for the 0.05 M Pd concentration for the Pd-γ-Al_2_O_3_ photocatalyst. On the other hand, the PdO-γ-Al_2_O_3_ photocatalyst containing 0.010 M Pd shows 10%, 40% and 52% degradation for BTB, MB and MO organic dyes, respectively, and their photocatalytic degradation increased to 26%, 46% and 68% upon increasing the Pd concentration to 0.05 M. These degradation results indicate that Pd-γ-Al_2_O_3_ NPs are more active in organic dye degradation compared with PdO-γ-Al_2_O_3_ NPs under the same experimental conditions. This can be explained by the Pd^0^ state having a higher electron–hole separation, thus enhancing the photon efficiency, which favors the migration of electrons onto the surface of the catalyst particles. This is due to the strong interaction of Pd^0^ with the organic dyes and also the high activity of Pd.

### Kinetics of organic dye degradation

**Fig. 7:**
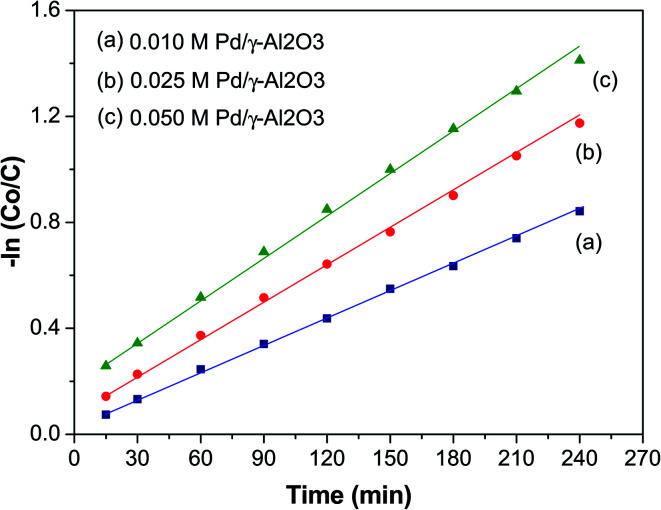
The kinetic plot of the photocatalytic degradation of BCG.

Fig. 7 shows a plot of −ln(*C*_0_/*C*) *versus* time (*t*, min) to study the kinetics of the photodegradation of BCG. From this plot it can be seen that the photocatalytic degradation of the organic dye BCG abides by pseudo-first-order kinetics of the Langmuir–Hinshelwood model,^50^ which has a linear relationship between −ln(*C*_0_/*C*) and *t*. The photocatalytic reaction may be depicted as:−ln(*C*_0_/*C*) = *kt*where *C*_0_ is the initial concentration of BCG, *C* is the concentration of BCG at time ‘*t*’, and the degradation rate constant is assigned as *k*. The value of *k* and the linear regression factor for the BCG dye are given in Table 2. The value of ‘*k*’ was calculated using the slope of the plot.

**Table 2:** Kinetic parameters for photocatalytic degradation of BCG dye

Initial conc. (*C*)	*k* (min^−1^)	*R* ^2^
0.010 M	0.0078	0.9969
0.025 M	0.0112	0.9951
0.050 M	0.0224	0.9918

The ‘*k*’ values decreased as the dye concentration increased, in agreement with the Langmuir–Hinshelwood model, which indicates that the reactant is first adsorbed onto the photocatalyst surface followed by degradation under photon irradiation. With the initial reactant concentration, the molecules are aggregated on the photocatalyst surface, which results in the excited molecules being quenched.^51^ Furthermore, high adsorption of the incident photons is due to the increase in the initial concentration, which results in a decrease of the reaction rate constant.^52^ The *R*^2^ values (Table 2) clearly show that the organic dyes are following a photocatalytic degradation that fits pseudo-first-order reaction kinetics.

### Effect of organic dye concentration

**Fig. 8:**
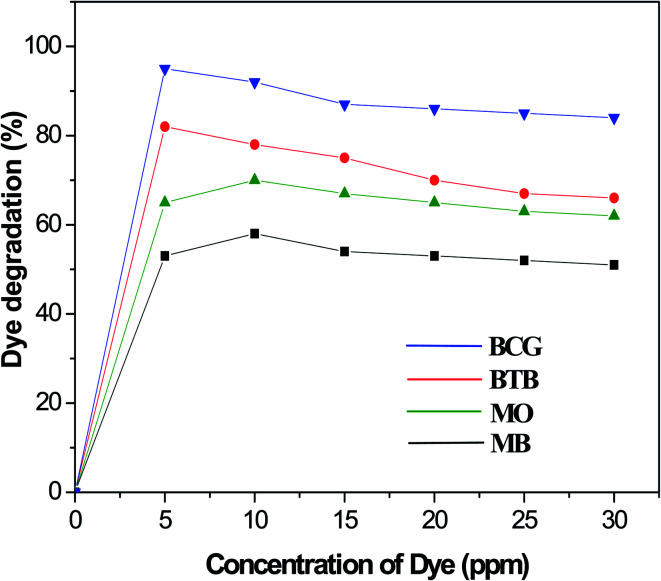
Degradation (%) of organic dyes at different concentrations.

The photocatalytic degradation of organic dyes also depends on the adsorption of the organic dye onto the surface of the photocatalyst, and only the amount of dye adsorbed onto the photocatalyst will participate. The effect of organic dye concentration on the photocatalytic degradation rate was investigated by examining the photodegradation at different concentrations of organic dye, including 5, 50, 10, 15, 20, 25 and 30 ppm. Fig. 8 shows the percentage degradation of the organic dyes under similar conditions of a fixed amount of photocatalyst and time of irradiation. The results indicate that, as the concentration increased, the degradation percentage decreased for all the Pd-γ-Al_2_O_3_ and PdO-γ-Al_2_O_3_ photocatalysts. The adsorption of organic dye molecules onto the photocatalyst surface affects its ability to absorb photons (light) and the subsequent generation of highly reactive radicals, thus having a significant effect on the percentage of photodegradation. This indicates that the adsorption of organic dye depends on its initial concentration. This is explained by the fact that, as the organic dye concentration increases, more organic substances are adsorbed onto the surface of the Pd-γ-Al_2_O_3_ and PdO-γ-Al_2_O_3_ photocatalysts, which allows fewer photons to absorb on the photocatalyst surface.^53^ This leads to a decrease in the formation of hydroxyl (˙OH) radicals, since the organic dye molecules occupy the active sites of the photocatalyst, which results in a lower percentage of degradation. In this work, for the organic dyes BCG and BTB, the highest degradation was obtained at a concentration of 5 ppm, whereas for the dyes MO and MB the highest degradation was achieved at 10 ppm concentration, and was further reduced on increasing the concentration of the dyes.

### Influence of the chemical structure of the organic dye on photodegradation

**Fig. 9:**
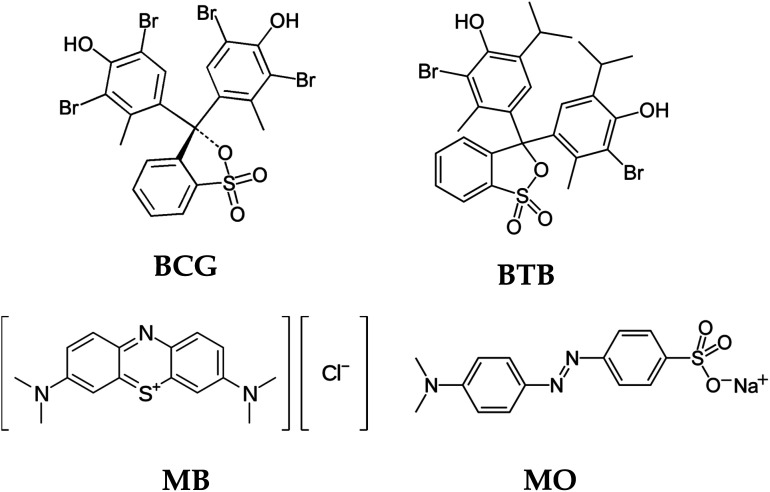
Structures of the organic dyes.

The chemical structure of the organic dyes influences the reactivity of the dye in the photocatalytic degradation reaction. The chemical structures of the organic dyes used in this study are presented in Fig. 9. Organic dye molecules usually contain different numbers of functional groups. This can lead to different types of reactions, releasing various intermediate products that affect degradation efficiency. The organic dyes BCG, BTB and MO possess one sulfonic group, which is a more powerful electron-withdrawing group. The organic dyes possessing this sulfonic group show almost the same reactivity with respect to oxidation by hydroxyl radicals.^54^ Furthermore, BCG and BTB have two hydroxyl groups in their structure, and these hydroxyl groups can intensify the resonance and, consequently, the degradation rate of the dye. The presence of sulfonic and hydroxyl groups in the structure of the organic dye molecules makes them more reactive in a photocatalytic degradation process. The organic dye MB shows less photodegradation compared to the other organic dyes. This is due to the presence of chloro and methyl groups in the MB dye molecules. The chloro group in the MB dye molecule may be substituted by hydroxyl radicals^54^ leading to the formation of chloride anions in the solution, and thus a decreased rate of degradation. Similar results were obtained in Wang's^55^ research on the photocatalytic degradation of eight commercial dyes with different structures containing chloro and sulfonic groups as substitute groups. These results indicate that chloride-substituted dyes are less reactive than sulfonic-substituted dyes in the photocatalytic process.

### Effect of pH on BCG degradation

**Fig. 10:**
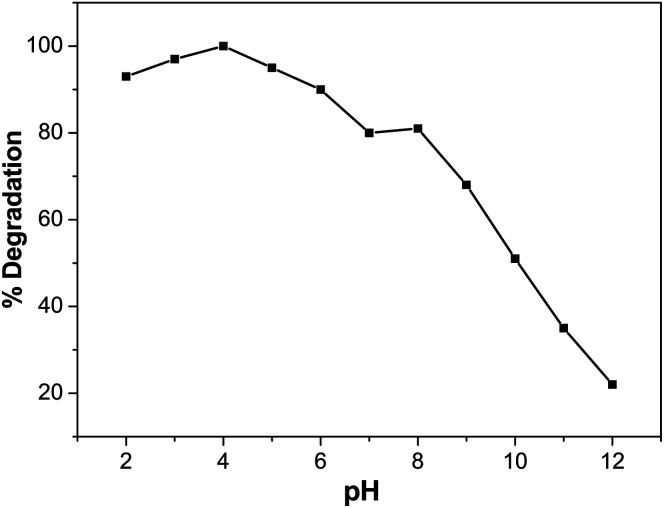
Effect of pH on degradation (%) of BCG.

In photocatalytic degradation, the pH is essential, since it plays a crucial role in the release of the proton and development of hydroxyl radicals. Different industries, such as dye manufacture and surface coating, discharge their effluent at varying stages into water reservoirs. Therefore, it is essential to evaluate the effect of pH on the pollutant photodegradation in the organic dye mechanism. This includes attack on the hydroxyl radical, direct electron reduction in the conducting band and direct oxidation by positive holes.^56^ The pH effect was achieved at different pH values with 5 ppm of dye solution and 0.01 M of photocatalyst. Diluted sodium hydroxide (NaOH) or hydrochloric acid (HCl) was used to change the pH of the solution. Fig. 10 indicates that pH has a role in the degradation of BCG. The photocatalytic degradation of BCG increased from pH 2 to 4 and then decreased up to pH 12. The maximum degradation was observed in acid medium and the optimum degradation of BCG was found at pH 4. The interaction of the photocatalyst ions and the positive pores of the catalyst led to the formation of a large number of hydroxyl radicals and the formation of a rich surface complex bond, as a result of efficient electron transfer.^57^ The photodegradation is likely to diminish at extreme pH values due to coulombic repulsion between the OH^−^ anions and the very positive charged oxide surface, induced by diffusion on the photocatalyst of the surface by the further generation of hydroxide ions. The increased pH may also induce a cathodic displacement of the Pd/PdO catalyst valence band position, leading to a decrease in the oxidation ability of the holes.^58^

### Mechanism for photocatalytic degradation

**Fig. 11:**
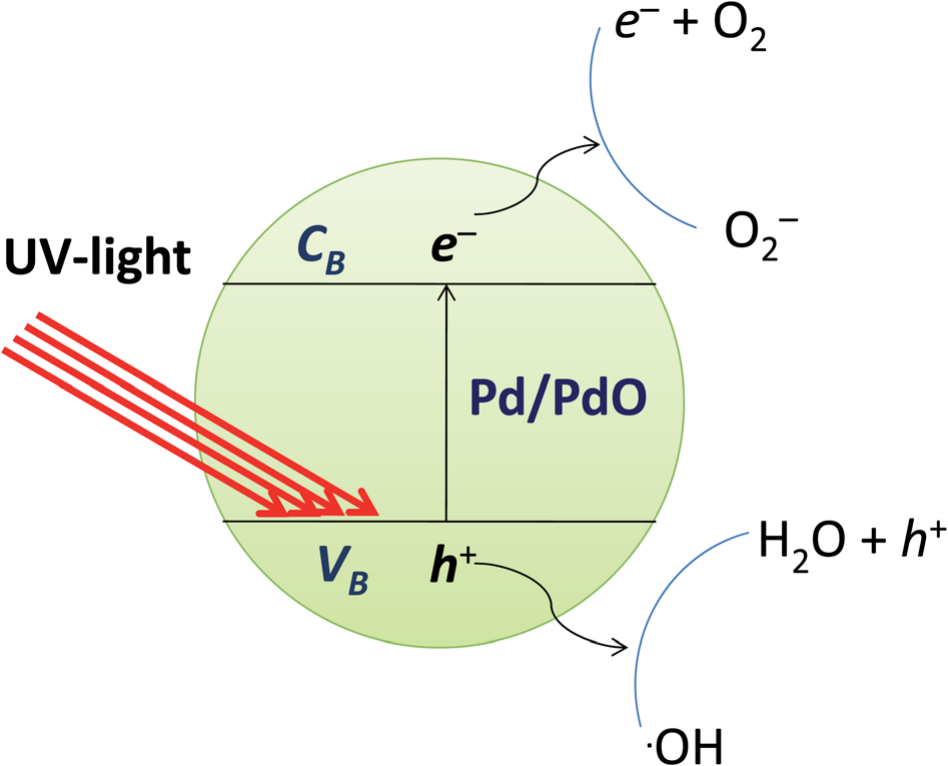
Mechanism of photocatalytic degradation of organic dyes by Pd-γ-Al_2_O_3_ and PdO-γ-Al_2_O_3_ catalysts.

The photocatalytic reaction is an advanced oxidative process conducted by a photocatalyst under the influence of light. Photocatalytic materials absorb photons with energy equal to or greater than the energy difference between the valence and conduction bands of the photocatalyst. The photocatalytic mechanism for the degradation of organic dyes from the Pd/PdO surface is defined as the formation of an electron conduction band and hole valence band pair on the photocatalyst, and their passage into the organic pollutants. The band gap energy of the Pd-γ-Al_2_O_3_ and PdO-γ-Al_2_O_3_ photocatalysts was obtained from the PL spectra and is between 2.5 and 2.7 eV, which is almost the same as that previously reported.^59^Fig. 11 shows the mechanism of photocatalytic degradation of the employed organic dyes using Pd-γ-Al_2_O_3_ and PdO-γ-Al_2_O_3_ NPs as photocatalysts. The UV light falling on the surface of Pd/PdO NPs absorbs photons with energy equal to or greater than the band difference between the photocatalyst's valence and conduction bands. The photon absorption allows the valence electrons (e^−^) to excite the path from the valence to the conduction band, resulting in positive holes (h^+^) in the valence band. The e^−^ and h^+^ play an important role in the formation of reactive radicals. The positive holes react with H_2_O to form radicals of hydroxyl (˙OH), whereas the excited electrons reduce the amount of adsorbed oxygen to give radical superoxide (˙O_2_^−^). These ˙OH and ˙O_2_^−^ reactive radicals take part in the process of organic dye photodegradation. The ˙OH free radicals are strong oxidizing species that attack the organic groups of the contaminant dyes on the photocatalyst surface^60^ and undergo different reactions to make non-hazardous organic contaminants or to transform them completely into carbon dioxide and water.^61^ However, the photogenerated electron and hole pairs are recombined, which affects the performance of the photocatalyst. Pd reduces the recombination of electron–holes produced by light and improves the photocatalytic reactions and efficiency of degradation. In order to facilitate photocatalytic reactions, the recombination of photogenerated charge carriers should therefore be reduced and controlled. In the following equations, the reaction mechanism for the photocatalytic degradation of organic dyes under UV light is given:Pd/PdO NPs → Pd/PdO NPs (h^+^ + e^−^)O_2_ + e^−^ → ˙O^2−^H_2_O + h^+^ → ˙OHBCG/BTB/MB/MO + ˙O^2−^ → degradable productsBCG/BTB/MB/MO + ˙OH → degradable products

### Evaluation of the recyclability of the photocatalysts

**Fig. 12:**
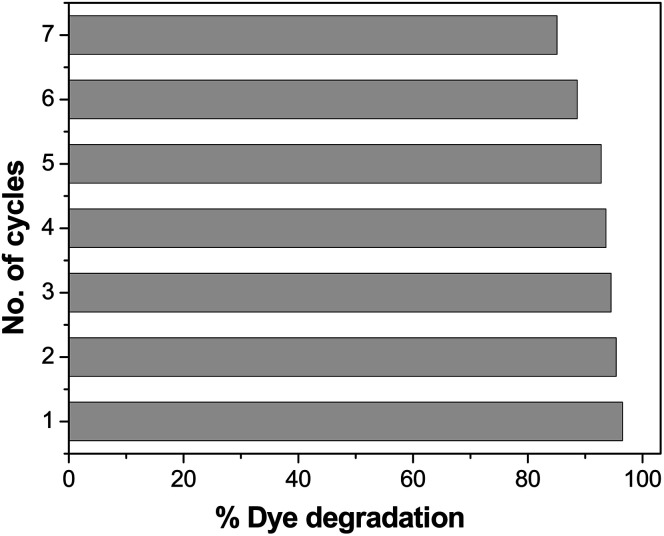
Loss of photocatalytic activity of Pd-γ-Al_2_O_3_ and PdO-γ-Al_2_O_3_ catalysts upon repeated cycles.

Recyclability is one of the most important features of a photocatalyst. The repeated performance of Pd-γ-Al_2_O_3_ and PdO-γ-Al_2_O_3_ NPs as nanocatalysts was examined by recycling the NPs. This can be done by washing and rinsing the photocatalysts with double-distilled water, then by drying them and using them again as photocatalysts under similar experimental conditions. The same photocatalysts and the same procedure were followed for seven repeated cycles. The catalytic activity of Pd-γ-Al_2_O_3_ and PdO-γ-Al_2_O_3_ photocatalysts on organic dye degradation does not show a noticeable variation for up to 5 cycles. After 5 cycles there was a slight decrease in efficiency, as shown in Fig. 12. This reduced catalytic activity might be due to deposition of photosensitive hydroxide on the surface of the Pd-γ-Al_2_O_3_ and PdO-γ-Al_2_O_3_ photocatalysts, which might block the reactive sites of the photocatalyst.

## Conclusions

Pd-γ-Al_2_O_3_ and PdO-γ-Al_2_O_3_ photocatalysts were successfully prepared by the chemical co-precipitation method. Both Pd-γ-Al_2_O_3_ and PdO-γ-Al_2_O_3_ NPs are effective for photocatalytic degradation of the organic dyes BCG, BTB, MB and MO in aqueous medium under UV-light irradiation. The Pd-γ-Al_2_O_3_ photocatalysts have been found to be more effective than the PdO-γ-Al_2_O_3_ photocatalysts. Complete photodegradation was observed for the organic pollutant BCG. The rate of degradation increased as the photocatalyst concentration and irradiation time increased. The possible mechanism of photocatalytic degradation of organic dyes by Pd-γ-Al_2_O_3_ and PdO-γ-Al_2_O_3_ catalysts was described. The catalytic activity of Pd and PdO NPs on the γ-Al_2_O_3_ surface can be explained by their capability of entrapping the photoinduced e^−^ and h^+^ to obtain more reactive ˙OH radicals. Moreover, the existence of Pd/PdO represses the recombination of light-generated e^−^ and h^+^ pairs and subsequently increases the photocatalytic efficiency. The recovered Pd-γ-Al_2_O_3_ and PdO-γ-Al_2_O_3_ photocatalysts also significantly degraded the organic dyes in aqueous medium, but their degradation efficiency was reduced after 5 cycles of use.

## Conflicts of interest

The authors declare that there is no conflict of interest.

## Supplementary Material
